# The Effect of Displacement Amplitude on Fretting Wear Behavior and Damage Mechanism of Alloy 690 in Different Gaseous Atmospheres

**DOI:** 10.3390/ma14195778

**Published:** 2021-10-02

**Authors:** Long Xin, Lanzheng Kang, Weiwei Bian, Mengyang Zhang, Qinglei Jiang, Tetsuo Shoji

**Affiliations:** 1 National Center for Materials Service Safety, University of Science and Technology Beijing, Beijing 100083, China; klzb20200474@163.com (L.K.); b20190454@xs.ustb.edu.cn (W.B.); tshoji@fri.niche.tohoku.ac.jp (T.S.); 2 China Nuclear Power Operation Technology Corporation, Ltd., Wuhan 430223, China; zhangmy02@cnnp.com.cn (M.Z.); jiangql@cnnp.com.cn (Q.J.); 3 Frontier Research Initiative, New Industry Creation Hatchery Center, Tohoku University, 6-6-10, Aramaki Aoba, Aoba-ku, Sendai 980-8579, Japan

**Keywords:** fretting wear, fretting regime, fretting-induced fatigue, fretting-induced wear, alloy 690, displacement amplitude

## Abstract

The effect of displacement amplitude on fretting wear behavior and damage mechanisms of alloy 690 in air and nitrogen atmospheres was investigated in detail. The results showed that in air, the friction coefficient gradually increased with the increase in displacement amplitude which conformed to the universal law. In nitrogen, however, it had the highest point at the displacement amplitude of 60 μm due to very strong adhesion. Whether in air or nitrogen, the wear volume gradually increased with the increase in displacement amplitude. The wear volume in air was larger than that in nitrogen except at 30 μm. At 30 μm, the wear volume in air was slightly smaller. With an increase in displacement amplitude, a transformation of fretting running status between partial slip, mixed stick-slip, and final gross slip occurred along with the change of Ft-D curves from linear, to elliptic, to, finally, parallelogrammical. Correspondingly, the fretting regime changed from a partial slip regime to a mixed regime to a gross slip regime. With the increase in displacement amplitude, the transition from partial slip to gross slip in nitrogen was delayed as compared with in air due to the strong adhesion actuated by low oxygen content in a reducing environment. Whether in air or nitrogen, the competitive relation between fretting-induced fatigue and fretting-induced wear was prominent. The cracking velocity was more rapid than the wear. Fretting-induced fatigue dominated at 30 μm in air but at 30–60 μm in nitrogen. Fretting-induced wear won the competition at 45–90 μm in air but at 75–90 μm in nitrogen.

## 1. Introduction

Fretting wear is mainly distinguished from sliding wear due to relatively small displacements. The small movements between two contact surfaces can cause fretting wear, which is typical damage of materials during friction and wear [1,2]. When the displacement amplitude changes, the fretting behavior and wear mechanism will vary accordingly. For fretting behavior, as the displacement amplitude changes, the fretting running status alters from partial slip to gross sliding [3]. Meanwhile, the fretting loop varies from linear to parallelogrammical [4]. Therefore, three fretting regimes, including the partial slip regime (PSR), mixed regime (MR), and gross slip regime (GSR), appear on fretting maps [5,6,7]. The partial slip regime usually occurs when the displacement amplitude is small. The typical cracking is often initiated at the interface between the adhesion zone and microslip zone [8]. Severe wear damage accompanied by oxidation occurs when the displacement amplitude is large in its gross slip regime. The fatigue cracking is suppressed and limited [1,9]. In a mixed regime, the typical wear damage in the partial slip regime and the gross slip regime has a concurrence that has many complexities to analyze [10].

Alloy 690 has been widely used as a material in heat exchanger tubes in nuclear power plants. Fretting wear between the tube and anti-vibration supporter can be induced by the flow-induced vibration [11]. In the field of fretting in SG tubing, materials such as alloy 690 and 600 and numerous factors like normal force [12,13,14], displacement amplitude [14,15,16,17], cycle number [18,19], vibration frequency [16], environment [20,21], material [22,23,24,25,26], water chemistry [11,27,28,29,30,31], and temperature [32] have been investigated. However, the fretting behavior and wear mechanism in a reducing environment, such as nitrogen, has not been fully understood. In the previous studies [20,33], we explored the formation mechanisms of nanostructured tribolayers at a given displacement amplitude. However, the effects of a wide range of displacement amplitude on the fretting wear in nitrogen have not been systematically studied. For a given material pair, the displacement amplitude is regarded as one of the major parameters because the fretting regime and wear damage can be transformed by changing the displacement amplitude [5,34,35].

The atmosphere composition is the environmental condition with the largest effect on fretting, which can influence the chemical reactions in the contacting surface and then alter the fretting response [36,37,38]. Lots of researchers noted the influences of oxygen, humidity, and atmosphere on fretting wear in different gases [36,37,38,39]. They showed that the wear debris played an important role in the fretting process under different environments. Therefore, we conducted the fretting experiments in an ambient atmosphere and a reduced environment (nitrogen) to explore whether the alloy 690 has a wide range of uses in some industries.

## 2. Materials and Methods

Alloy 690 and Type 304 stainless steel (304SS) (Jiuli Group Co., Ltd., Huzhou, China) were used in this study. The 304SS was used as an anti-vibration component [40] in the CANDU nuclear power plant. The diameter of the 304SS ball was 10 mm. The specimen size of alloy 690 was 10 × 10 × 1.09 mm^3^. The surface roughness (Ra), hardness, and diameter of the 304SS ball were 0.4 μm, 200 HV, and 10 mm, respectively. The alloy 690 plate was polished to a surface roughness of 0.04 μm with a hardness of ~235 HV. The chemical compositions and mechanical properties of these two materials are shown in Table 1 and Table 2, respectively.

A commercial fretting test rig (Optimol SRV-IV) (Optimol, Munich, Germany) with a ball-on-flat configuration was used in this research. The corresponding schematic diagram is shown in our previous studies [33]. The friction coefficient (FC) and friction force (Ft)-displacement (D) curves can be recorded online in real-time. Fretting wear tests were carried out three times for each condition in order to guarantee repeatability. A normal load of 100 N, a frequency of 20 Hz, a time of 30 min, and a displacement amplitude from 30 to 90 μm were selected as the test parameters. The test environments are at 320 °C in both air and nitrogen. The normal force of 100 N was chosen because it is slightly higher than the actual normal force of 10–90 N [27,41] in steam generator tubes. The test temperature of 320 °C was selected due to the actual operating temperature of 270–330 °C [42].

Laser scanning confocal microscopy (LSCM, Olympus LEXT OLS4000, Olympus, Tokyo, Japan) was used to detect the cross-sectional profiles and wear volumes of wear scars. Scanning electron microscopy (SEM, Zeiss Auriga, Carl Zeiss, Oberkochen, Germany), accompanied by an energy dispersive spectroscopy (EDS), was utilized to observe the wear surface and subsurface. Before each test and measurement, the specimens of alloy 690 were acoustically cleaned in acetone, then alcohol for 10 min, and, finally, dried in compressed air.

## 3. Results

**Figure 1:**
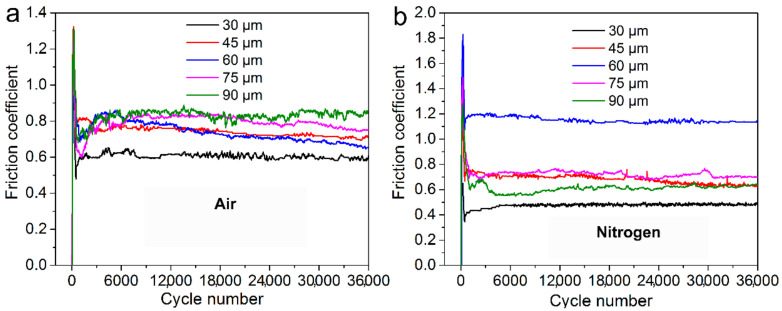
Friction coefficient of alloy 690 during fretting wear at different displacement amplitudes in (**a**) air and (**b**) nitrogen.

Figure 1a,b show the curves of the friction coefficient of alloy 690 during fretting wear at different displacement amplitudes in air and nitrogen, respectively. As shown in Figure 1a, the friction coefficient in air obviously goes up during the starting period of fretting then drops to a stable state with larger cycle numbers. As shown in Figure 1b, the variation of the friction coefficient in nitrogen is similar to that in air.

**Figure 2:**
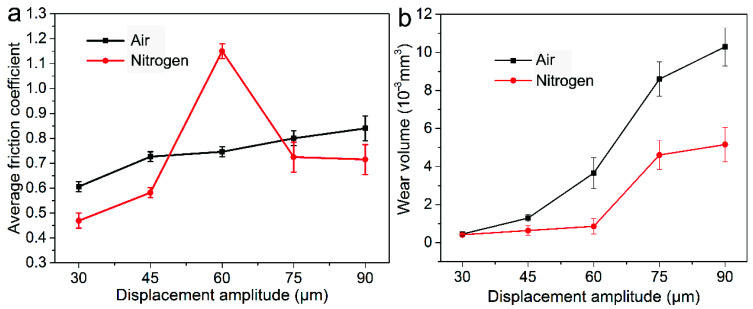
(**a**) The average friction coefficient and (**b**) wear volume of alloy 690 during fretting wear at different displacement amplitudes in air and nitrogen. The error bar is calculated by the standard deviation of three measurements.

Figure 2a shows the average friction coefficient of alloy 690 during fretting wear at different displacement amplitudes. In air, the average value gradually increases with increasing displacement amplitudes. In nitrogen, it gradually increases from 30 μm to 60 μm then decreases. The average value in air is larger than that in nitrogen except for the condition at 60 μm. Figure 2b shows the wear volume of alloy 690 during fretting wear at different displacement amplitudes. Whether in air or nitrogen, the wear volume gradually increases. The value of wear volume in air is larger than that in nitrogen except for the condition at 30 μm. At 30 μm, the wear volume in air is slightly smaller.

**Figure 3:**
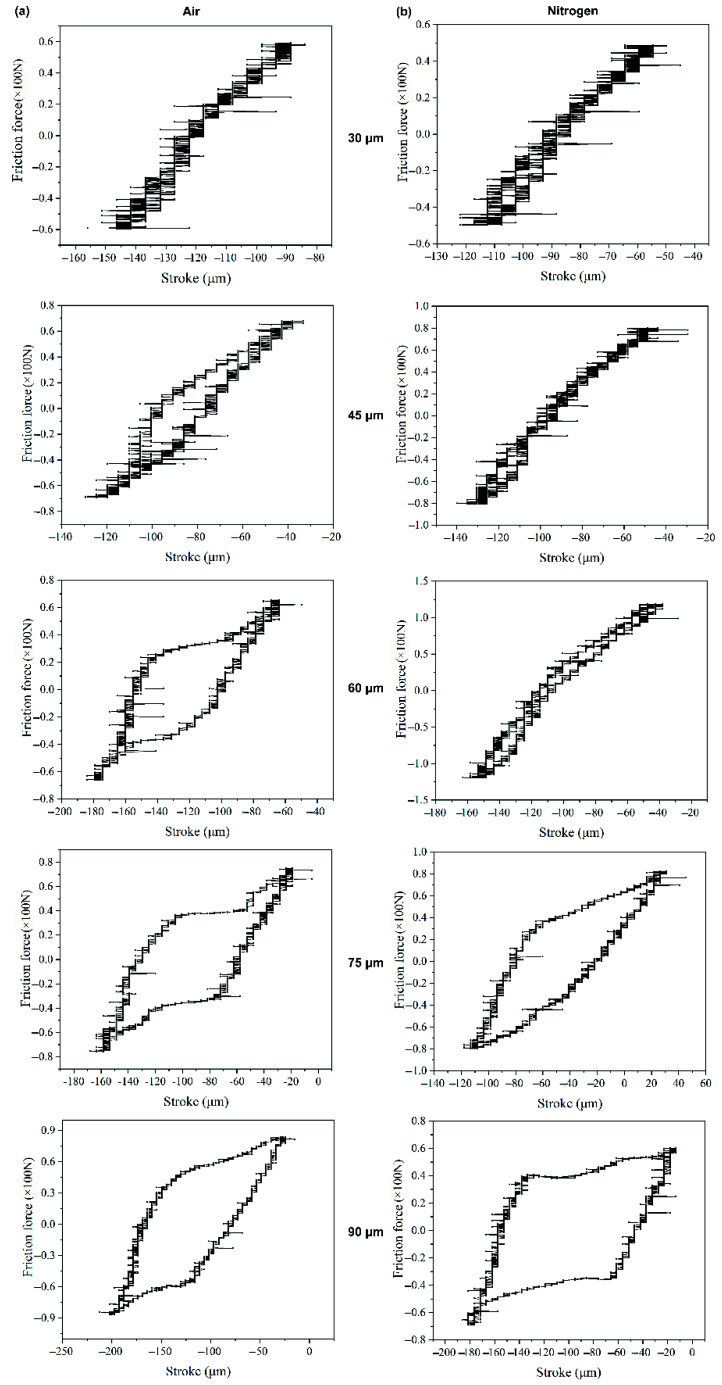
Ft-D curves of alloy 690 during fretting wear at different displacement amplitudes in (**a**) air and (**b**) nitrogen.

Figure 3 shows the Ft-D curves of alloy 690 during fretting wear at different displacement amplitudes in air and nitrogen, respectively. In air, the transition from a line to an ellipse then to a parallelogram is observed with the increase in the displacement amplitude (Figure 3a). In nitrogen, Ft-D curves remain linear when the displacement amplitude is not larger than 60 μm. Then, the transition from ellipse to parallelogram occurs when the displacement amplitude increases.

**Figure 4:**
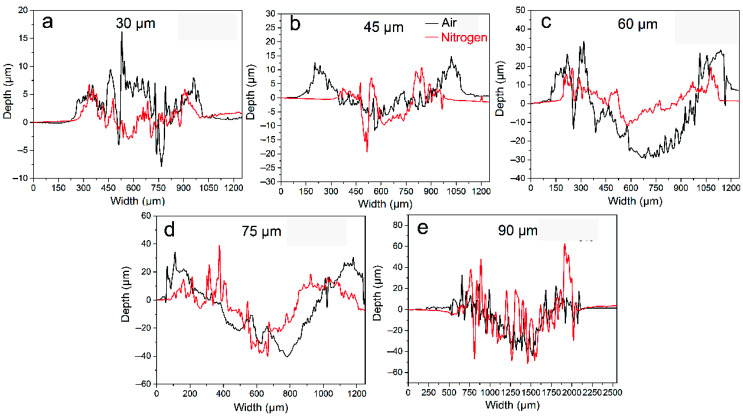
The typical cross-sectional profile of worn scars of alloy 690TT at the displacement amplitude of (**a**) 30 μm, (**b**) 45 μm, (**c**) 60 μm, (**d**) 75 μm, and (**e**) 90 μm.

Figure 4 shows the typical cross-sectional profile of worn scars of alloy 690 at different displacement amplitudes. As mentioned previously, the volume below the original surface was acknowledged as the wear volume or material loss, while the volume above the original surface was the transfer volume or material transfer [43]. At 30 μm, the width of the worn scar in air and nitrogen is basically the same. However, the material transfer in air is more obvious than that in nitrogen (Figure 4a). When the displacement amplitude is larger than 30 μm, the width of the worn scar in air is slightly larger than that in nitrogen (Figure 4b–e).

**Figure 5:**
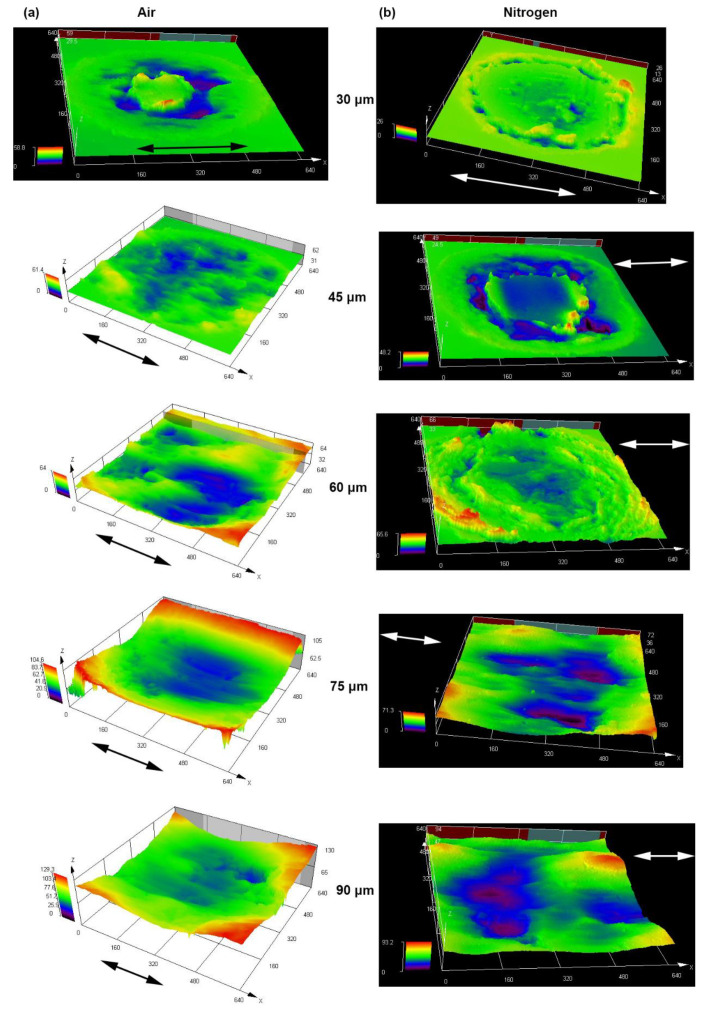
Three-dimensional-profile micrographs of alloy 690 during fretting wear at different displacement amplitudes in (**a**) air and (**b**) nitrogen. The arrows indicate the fretting direction.

Figure 5 shows the 3D-profile micrographs of alloy 690 during fretting wear at different displacement amplitudes in air and nitrogen. It can be found that whether in air or nitrogen, the wear width and depth gradually increased with the increase in the displacement amplitude. Compared with the situation in air, the wear width and depth in nitrogen were smaller.

**Figure 6:**
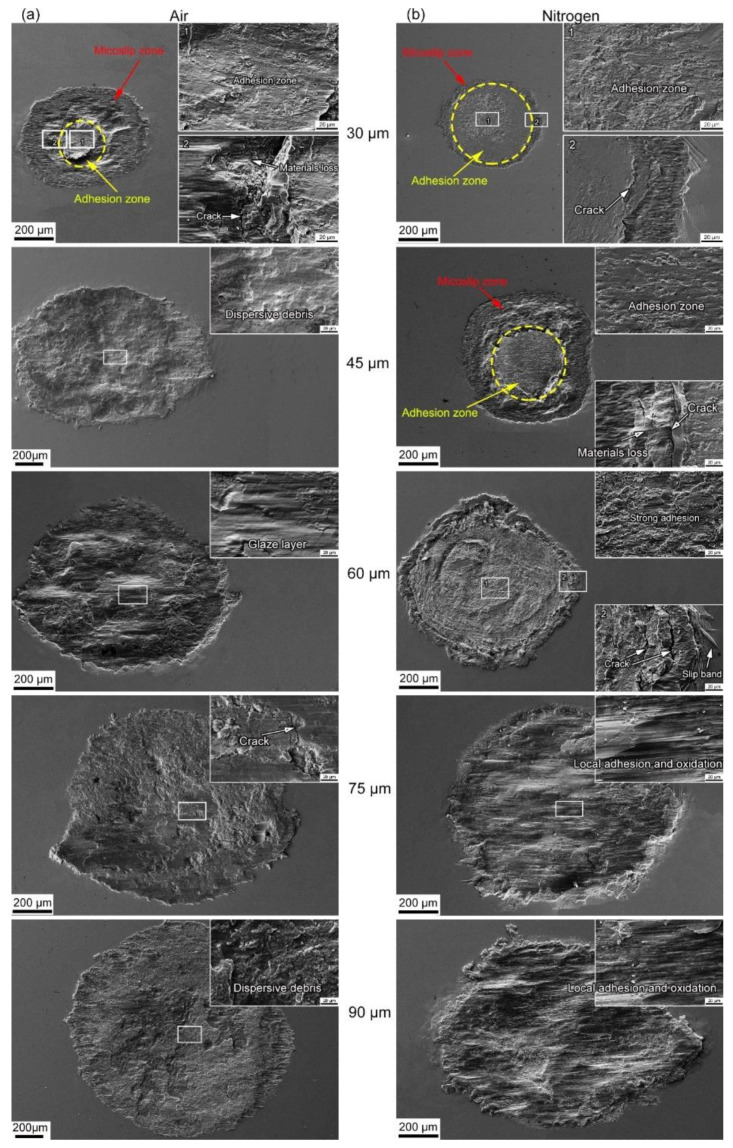
The SEM images of wear surfaces in alloy 690 during fretting wear at different displacement amplitudes in (**a**) air and (**b**) nitrogen.

Figure 6a,b show the SEM images of wear surfaces in alloy 690 during fretting wear at different displacement amplitudes in air and nitrogen, respectively. At 30 μm, fretting runs in the partial slip regime whether in air or nitrogen, which is indicated by the adhesion region in the center and the microslip region on the edge of the wear surface. In the adhesive zone, the interface is maintained under stick contact conditions by the adhesively joined asperities that will be plastically sheared along the fretting direction, as demonstrated by some tracks and the high-magnification micrograph in position 1. At the contact edge, a slight wear phenomenon is observed, as shown in position 2. However, in nitrogen, the area of the adhesion region is larger, while the area of the microslip region is smaller than in air. There are cracks between the adhesion regions and microslip regions in air and nitrogen. Furthermore, in air, material loss can be also found between the adhesion region and microslip region. At 45 μm, the adhesion region disappears in air but still exists in nitrogen. In nitrogen, the area of the adhesion region decreases while the area of the microslip region increases compared with that at 30 μm. At 60 μm in air, the glaze layer is formed on the wear surface. However, in nitrogen, strong adhesion still exists, and a strong plastic deformation can be found in the center. Cracks can be also found on the edge of the wear surface. At 75 μm, the wear surface in air is covered with a wear debris layer with cracks. In nitrogen, local adhesion can be found on the wear surface. The dark color indicates that the wear surface has been oxidized, while the light color indicates that the wear surface has not been oxidized. Furthermore, as shown in the high-magnification micrograph, the area of light color has the feature of adhesive tear marks to indicate the local adhesion. At 90 μm, the distribution of wear debris in air is dispersed. In nitrogen, local adhesion can also be found on the wear surface, which is similar to that at 75 μm.

**Figure 7:**
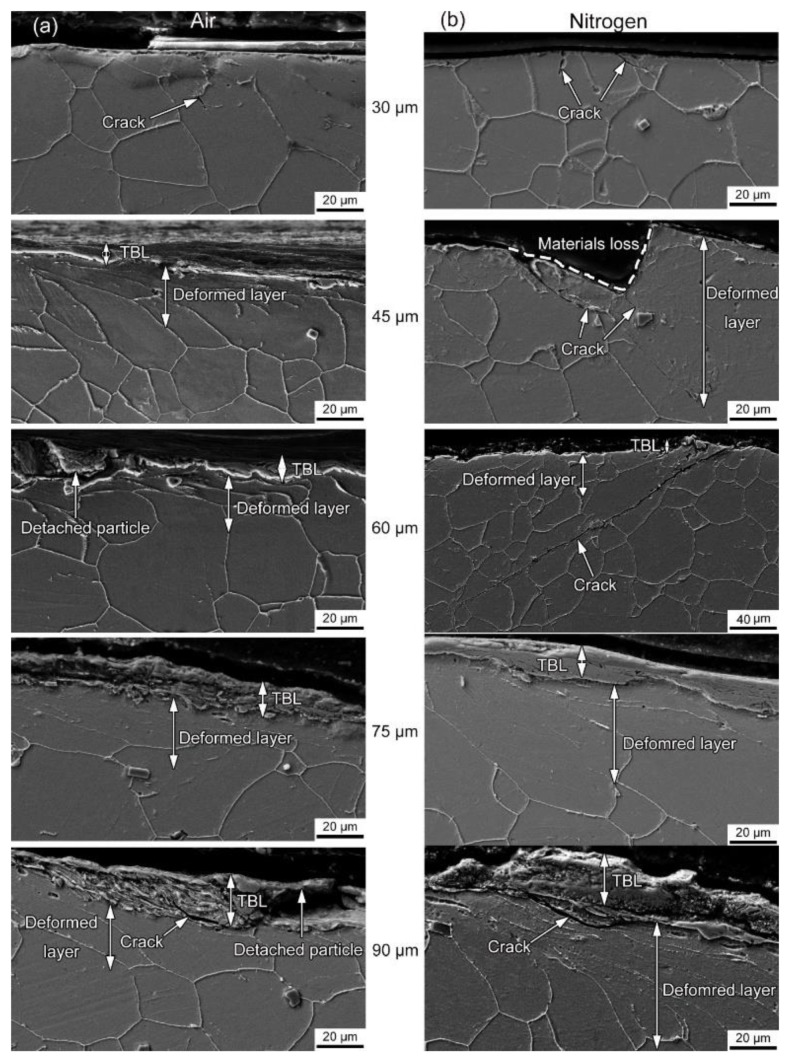
The SEM images of wear sub-surfaces in alloy 690 during fretting wear at different displacement amplitudes in (**a**) air and (**b**) nitrogen.

Figure 7a,b show the SEM images of wear sub-surfaces in alloy 690 during fretting wear at different displacement amplitudes in air and nitrogen, respectively. At 30 μm in air, a fatigue crack with a length of 25 μm between the adhesion region and microslip region can be found. The angle between the crack and the wear surface is approximately 45°. In nitrogen, two fatigue cracks are found in the cross-section. One of the cracks is almost straight to the deep subsurface. The other has an angle of 30° to the wear surface. At 45 μm in air, a third body layer (TBL) with a thickness of 6 μm is the outermost layer. Below it, there is a deformed layer with a thickness of 6 μm. There is no crack in the cross-section of these layers. However, in nitrogen, a fatigue crack with a length of 75 μm between the adhesion region and microslip region can be found. The angle between the crack and wear surface is approximately 45°. There is a deformed layer with a thickness of 65 μm near the region of material loss. At 60 μm in air, the thickness of the third body layer increases to 8 μm, accompanied by the detached particles. However, the thickness of the deformed layer decreases to 20 μm. In nitrogen, a fatigue crack with a length of 180 μm can be found between the adhesion region and microslip region. The angle between the crack and wear surface is approximately 40°. There is a deformed layer with a thickness of 45 μm in the cross-section. At 75 μm in air, the thickness of the third body layer increases to 15 μm. However, the thickness of the deformed layer is about 22 μm. In nitrogen, the fatigue crack disappears. The thickness of the third body layer increases to 13 μm. There is a deformed layer with a thickness of 42 μm in the cross-section. At 90 μm in air, the thickness of TBL further increases to 21 μm along with many cracks accompanied by more cracks. The thickness of the deformed layer is about 23 μm. In nitrogen, the thickness of the third body layer increases to 22 μm. There is a deformed layer with a thickness of 50 μm on the subsurface along with cracks.

**Figure 8:**
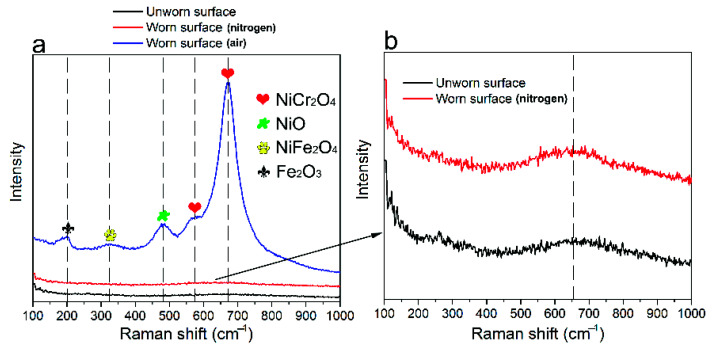
(**a**) Raman spectroscopy for the worn scars of alloy 690 during fretting wear at the displacement amplitude of 60 μm in air and nitrogen. (**b**) The magnified image of the curves from (**a**).

Figure 8 shows the Raman spectroscopy for the worn scars of alloy 690 during fretting wear at the displacement amplitude of 60 μm in air and nitrogen. The highest peak represents the major oxide existing on the wear surface. As shown in Figure 8a, in air, the main feature of a Raman shift of 678 cm^−1^ is observed, indicating NiCr_2_O_4_ [13]. The weak ones appearing at 203, 324, 480, and 576 cm^−1^ represent Fe_2_O_3_, NiCr_2_O_4_, and NiO, respectively [13,43,44]. Figure 8b shows the magnified image for the curves from Figure 8a. It can be found that the Raman spectra on worn and unworn surfaces are the almost same. The highest peak of a Raman shift of 658 cm^−1^ can be observed, indicating Fe_3_O_4_ [45].

## 4. Discussion

As shown in Figure 1, in the early stage of fretting, the original surface film was gradually destroyed. Then, direct metal–metal contact occurred, which resulted in the rapid increase in the friction coefficient due to the surface adhesion and plastic deformation at the contact point [46,47]. The wear debris would be formed after continuous surface work hardening caused the particles to peel off. A large amount of wear debris accumulated to form a third body layer, which was regarded as a solid lubricant to avoid metal–metal contact [48,49]. Therefore, the FC gradually decreased to a stable value along with the evolution of the third body layer. As shown in Figure 1 and Figure 2, in nitrogen, slightly lower friction and lower wear volume were measured as compared with that in air, which was similar to that in [36]. The specimens in the nitrogen atmosphere had lower wear, suggesting that oxidation is the primary wear mechanism in ball-on-plate contact or at least the most significant one. The reducing atmosphere (nitrogen) acted as a limit condition since nitrogen minimizes/eliminates oxidation [37]. However, at 60 μm in nitrogen, the friction was the highest due to the very strong adhesion without oxidation shown in Figure 8. The friction coefficient at the displacement amplitude of 30 μm was due to the mostly stick phenomena, which was in agreement with the linear Ft-D curves shown in Figure 3a,b, and it always occurred under a relatively low slip amplitude resulting from the oscillation accommodated by the elastic deformation. An increase in the friction coefficient occurred due to a gradual transition from elastic domination to local plastic deformation [10,35], as shown by the transition of Ft-D curves from linear to elliptic in Figure 3. With a further increase in displacement amplitudes, the Ft-D curves changed from ellipsis to parallelograms. Correspondingly, the increase in the friction coefficient with increasing displacement amplitudes was due to the change in wear mode from mixed stick-slip to gross slip [34,50].

For tangential fretting wear, Ft-D curves between contact surfaces are the most basic and important information. According to the definition method of fretting running status proposed by Zhou and Vincent [10,35,51], fretting ran in a partial slip regime (PSR) when Ft-D curves kept a linear shape with the relative motion coordinated by elastic deformation. Fretting located at mixed regime (MR) when the Ft-D curves were transformed to the ellipse were due to the elastic-plastic deformation. The gross slip regime (GSR) was achieved when all Ft-D curves changed to be parallelogrammical shapes. According to the above analysis, with the increasing displacement amplitude, a transition from partial slip to mixed stick-slip to final gross slip occurred gradually in air and nitrogen, as shown in Figure 3. Obviously, the transition from partial slip to gross slip in nitrogen was delayed when compared with that in air. This was due to the strong adhesion actuated by low oxygen content in the nitrogen environment.

With the increasing displacement amplitude, the transformation of the fretting running status from the partial slip regime to the mixed fretting regime to the gross slip regime occurred. Correspondingly, the fretting-induced fatigue cracking (FIFC) was the main damage mechanism in the partial slip regime and mixed fretting regime, while the fretting-induced wear (FIW) dominated in the gross slip regime [52]. The competition between fretting-induced fatigue cracking and fretting-induced wear was accompanied by the whole process of fretting wear. At 30 μm in air, the fatigue cracks wear observed at the interface of the stick-slip region shown in Figure 6a and Figure 7a due to the high shear stress concentration at the interface [53]. The adhesive wear in the adhesion zone and the abrasive wear in the microslip region were the dominating damage mechanisms. However, the FIFC was absolutely dominant because the fretting-induced wear was slight. In nitrogen, as the displacement amplitude increased from 30 to 60 μm, the length of the fatigue crack gradually increased, indicating the aggravated FIFC as shown in Figure 6 and Figure 7. Furthermore, the inclination angle in nitrogen was larger than that in air, which suggested the FIFC in nitrogen was more severe. As the displacement amplitude gradually increased, no crack could be found. Meanwhile, only some parallel cracks induced in delamination wear appeared, as shown in Figure 6 and Figure 7. This showed that the wear rate was more rapid than the FIFC, and fretting-induced wear won the competition. In the gross slip regime, whether in air or nitrogen, a stable and compacted layer on the top surface was known as the glaze layer (GL) with its stabilized high lubrication properties to lower the friction coefficient and wear volume, as shown in Figure 6 [54]. The formation of the glaze layer was dependent on time and temperature [55]. As the displacement amplitude increased, the glaze layer could be broken by larger stress and strain, as shown in Figure 6 and Figure 7.

## 5. Conclusions

In this study, the effect of displacement amplitude on fretting wear behavior and damage mechanisms of alloy 690 in air and nitrogen atmospheres was investigated in detail. Based on the above analysis, the following conclusions are obtained:

1. In air, the friction coefficient gradually increased with the increase in the displacement amplitude, which conformed to the universal law. In nitrogen, however, it had the highest point at 60 μm due to very strong adhesion. Whether in air or nitrogen, the wear volume gradually increased with the increase in the displacement amplitude. The wear volume in air was larger than that in nitrogen except for the condition at 30 μm. At 30 μm, the wear volume in air was slightly smaller.
2. With the increase in displacement amplitude, a transition from partial slip to mixed stick-slip to final gross slip occurred along with the change of Ft-D curves from linear to elliptic to, finally, parallelogrammical. Correspondingly, the fretting running status changed from partial slip regime to mixed regime to gross slip regime. With the increase in the displacement amplitude, the transition from partial slip to gross slip in nitrogen was delayed as compared with air due to the strong adhesion actuated by low oxygen content in a reducing environment.
3. Whether in air or nitrogen, the competitive relation between fretting-induced fatigue and fretting-induced wear was prominent. The cracking velocity was more rapid than wear, and fretting-induced fatigue dominated at 30 μm in air but at 30–60 μm in nitrogen. Fretting-induced wear won the competition at 45–90 μm in air but at 75–90 μm in nitrogen.

## Figures and Tables

**Table 1:** Chemical composition of alloys 690TT and 304SS (wt.%).

Specimen	Element								
	Ni	Fe	Cr	C	Ti	Mn	Si	P	S
alloy 690	Bal	11.6	29.9	0.025	0.30	0.25	0.33	0.086	0.0025
304SS	9.35	Bal	18.3	0.018	–	1.31	0.31	0.034	0.0025

**Table 2:** Mechanical property of the alloys.

Specimen	Vickers Hardness (HV)	Yield Strength (MPa)	Tensile Strength (MPa)
alloy 690	235	325	725
304SS	210	265	595

## Data Availability

The data presented in this study are contained within the article.
